# Supplementary material for: Healthy Diet-Related Knowledge, Attitude, and Practice (KAP) and Related Socio-Demographic Characteristics among Middle-Aged and Older Adults: A Cross-Sectional Survey in Southwest China
Source: Nutrients. 2024 Mar 17;16(6):869. doi: 10.3390/nu16060869 (PMC10974890; doi:10.3390/nu16060869)
Supplement: Supplementary file 1 [file nutrients-16-00869-s001.zip › File S1. Questionnaire on Dietary Knowledge, Attitude and Behavior of Southwest China Residents.pdf]

Questionnaire Code: Survey Date: Survey Location: Surveyor:

Questionnaire on Dietary Knowledge, Attitude, and Behavior of Southwest China Residents

Informed Consent Form

Dear Southwest China Residents, in order to further understand the impact and influence of regional dietary cultural differences and consumption behavior on public health, this study will conduct a questionnaire survey among residents in the Eastern, Southern, Central, Northern, Northwestern, Southwest, and Northeast regions of China. Through your truthful answers, we hope to understand the dietary health status of the general public, in order to support the implementation of the "Chinese Residents' Dietary Guidelines (2016)" nutrition policy and promote the formation and inheritance of a reasonable diet and a new healthy dietary culture in our country.

This project has no commercial significance or purpose, and your answers are not right or wrong. We will use an anonymous approach that will not have any impact on the respondents. Respondents have the right to raise questions or withdraw at any time. (This survey will take approximately 5-10 minutes of your time. We hope for your cooperation and support.)

Thank you for your cooperation!

China Nutrition Society - Diet Behavior and Diet Culture Survey Research Team

Informed Consent Signature: I have read and understood this informed consent for the survey. I voluntarily participate.

Respondent's Signature:

Section 1: Basic Information

1.Gender: A Male B Female

2.Date of Birth:_____ Year_____Month_____Day

3.Your height is:_____ Centimeters, for example, 170 cm

4.Your weight is:_____ Kilograms, for example, 60 kg

5.Your ethnicity: A Han B Other ethnicities____

6.Your current place of residence:_____ Province/Municipality_____District/County A Rural B Urban

7.Are you a local resident of the above-mentioned place who has lived there for generations?

A Lived here for generations (skip to question 9)

B Moved from another place

8.If you moved from another place, how many years have you lived in the current place?
A Less than 1 year B 1-2 years C 3-5 years D 6-9 years E 10 years and above

9.What is your occupation:
A Agricultural/Fishery/Livestock Worker/Factory Worker/Driving Instructor/Fitness Instructor/Police Officer/Manual Laborer, etc.

B Student

C Self-employed/Contractor/Field Reporter

D Freelancer/Retired

E Teacher/Ordinary Employee (Office/Office Building Worker)/Government, Organization Official/Public Servant/Doctor/Lawyer/Arts and Sports/Enterprise Manager (including grassroots and mid-to-senior level managers)

F Salesperson/Store Clerk/Waiter/Security Guard/Hairdresser

G Other occupation (please specify)_____

10.Your education level:
A Elementary school and below B Junior high school C High school/Vocational school/Technical school D Junior college E Bachelor's degree F Master's degree and above

11.In the past year, how many people live together in your family (including yourself)?______

12.What is the average total monthly income of your family (including income from sources such as housing leasing)?

A Below 3000 yuan B 3000-4999 yuan C 5000-9999 yuan D 10,000-19,999 yuan E 20,000-39,999 yuan F Above 40,000 yuan

13.From which channels do you obtain nutrition-related knowledge? (Multiple choices possible)
A Expert lectures/training B Books, newspapers, magazines C Radio, television D Family and friends or WeChat Moments

E Internet, public accounts, TikTok videos, etc. F Health product salesperson's explanation

14.Have you ever had or experienced the following diseases or symptoms? (Multiple choices possible)
A Hypertension B Hyperlipidemia C Diabetes D Gout E None

Section 2: Knowledge Part (1 liang = 50 grams ≈ 1 normal-sized egg)

1.According to the "Chinese Residents' Dietary Guidelines (2016)", how many grams of cooking oil are recommended for daily consumption by adults?
A 5-10 grams B 25-30 grams C 40-50 grams D No limit E Not sure

2.According to the "Chinese Residents' Dietary Guidelines (2016)", what is the maximum daily salt intake recommended for adults?
A 3 grams B 6 grams C 10 grams D No limit E Not sure

3.According to the "Chinese Residents' Dietary Guidelines (2016)", how many grams of dairy and dairy products are recommended for daily consumption by adults?
A 100 grams B 200 grams C 300 grams D No limit E Not sure

4.According to the "Chinese Residents' Dietary Guidelines (2016)", how much water should adults consume daily? (1 bottle of mineral water is approximately 550ml)
A 1000-1200ml B 1500-1700ml C More than 2000ml D No limit E Not sure

5. According to the "Chinese Residents' Dietary Guidelines (2016)", what is the recommended daily intake of vegetables for adults?
A 100-200 grams B 200-300 grams C 300-500 grams D No limit E Not sure

6. According to the "Chinese Residents' Dietary Guidelines (2016)", what is the recommended minimum number of food groups for adults?

A 10 groups B 12 groups C 15 groups D 20 groups E Not sure

7. What is the best way to supplement calcium?

A Spinach B Bone soup C Rice D Milk E Not sure

8. If your breakfast consists of 1 boiled egg, 1 cup of milk, and 1 plate of vegetables, what other food do you think you need to choose?

A 1 steamed bun B 1 deep-fried dough stick C 1 plate of pickles D 1 steak E Not sure

9. Compared to traditional communal dining, which of the following is not an advantage of individual portion dining?

A Reducing the risk of oral transmission of diseases (such as reducing the spread of Helicobacter pylori) B Controlling food intake
C Facilitating emotional communication D Reducing food waste E Promoting personalized diets F Not familiar with individual portion dining

10. Which of the following is most directly and closely related to the occurrence of hypertension in diet?

A Salt B Sugar C Oil D Vinegar E Not sure

11. How long is it generally safe to consume pickled vegetables?

A 2-3 days B About 1 week C About 2 weeks D About 3 weeks E Not sure

12. If drinking alcohol, adult males should not exceed how many bottles of beer per day? (1 bottle is about 500ml)

A 1 bottle B 1.5 bottles C 2 bottles D 3 bottles E Not sure

13. If drinking alcohol, adult females should not exceed how many bottles of beer per day? (1 bottle is about 500ml)

A 1 bottle B 1.5 bottles C 2 bottles D 3 bottles E Not sure

14. Which of the following foods, when consumed in moderation, helps protect the cardiovascular system?
A Fish B Chicken C Pork D Beef E Lamb F Not sure

15. Which of the following foods can gout patients choose?
A Beer B Pork liver C Bone (meat) soup D Eggs E Not sure

16. What is the best source of iron?
A Animal organs B Milk C Spinach D Stir-fried dishes in an iron pot E Not sure

17. Long-term tea drinking helps prevent cardiovascular and cerebrovascular diseases.
A True B False C Not sure

18. Which of the following statements about consuming animal organs is correct? (Multiple choices allowed)
A Liver is rich in vitamin A B Animal organs should be consumed at least 2-3 times a month C Animal organs have higher cholesterol content D Animal organs belong to high purine foods E Animal organs contain more toxins

Section 3: Attitude Part (Please select the option that best reflects your attitude towards the statements in the question)

Items	Strongly agree	Agree	Neutral	Disagree	Strongly disagree
1.Eating wild animals is more nutritious.					

2.A vegetarian diet is more beneficial for physical health than a meat-based diet.					
3.It is sufficient to eat until about 70-80% full in daily meals.					
4.Eating takeaway food does not have an impact on health.					
5.Traditional Chinese medicine for health preservation is very effective and should be fully retained.					
6.During holidays and festivals, satisfying one's cravings is more important than considering health issues.					
7.During holidays and festivals, satisfying one's cravings is more important than considering health issues.					
8.Sugary drinks have a better taste and can be used as a substitute for plain water.					
9.Red foods like brown sugar can replenish blood.					
10.Red foods like brown sugar can replenish blood.					
11."No feast without alcohol," it is necessary to drink alcohol to entertain guests.					
12.Smoking while eating.					
13.What is your opinion on the behavior of forcibly persuading others to drink (such as using statements like "If you don't drink, it means you look down on me")?					

14.The concept of food compatibility and incompatibility.					
15.Homemade fermented foods (such as homemade alcohol/tofu) are healthier than commercially processed ones.					
16.When cooking, use vegetable oil to replace some animal fat (such as lard).					
17.When a restaurant's food tastes good, it is not necessary to pay too much attention to the environment.					
18.Seasonings other than salt (such as chicken essence, monosodium glutamate, soy sauce) also increase salt intake.					
19.Pay attention to the balance between coarse and refined grains in staple foods (i.e., whole grains and refined white flour).					

Section 4: Dietary Behavior

1.What is the taste preference of your usual diet?

A Light　B Sour　C Sweet　D Spicy　E Salty

2.Where do you usually eat your meals?

A At home　B Cafeteria at work　C Restaurant　D Fast food chain　E Food stalls/Street vendors

3.Where do you mainly buy groceries?

A Large supermarket　B Convenient store near home　C Farmers' market　D Roadside vendors　E Online shopping　F Homegrown　G Never buy groceries

4. Please fill in the following information based on your actual situation:

Items	Never	Occasionally	Sometimes	Often	Always
(1) Pay attention to nutrition labels when purchasing pre-packaged foods.					
(2) Separate raw and cooked ingredients when handling food.					
(3) Frequency of eating out (including takeaway, excluding meals at home and cafeteria)					
(4) Take leftover food home when dining out.					
(5) Encourage drinking during social gatherings with friends to liven up the atmosphere.					
(6) Frequency of washing hands before meals.					
(7) Frequency of consuming wild game (wild animals).					

5. How often do you consume foods prepared using the following cooking methods (including homemade and restaurant-prepared foods)? Please mark " √ " in the appropriate column:

Items	Never	Occasionally	Regularly	Frequently	Always
(1) Stir-fried, sautéed, or stir-fried foods (e.g., stir-fried vegetables)					
(2) Steamed, boiled, blanched, or stewed foods					

(3) Pan-fried, deep-fried, or grilled foods					
(4) Cold dishes or raw foods					

6.How often do you have breakfast in a week?
A Never B 1-2 times C 3-4 times D 5-6 times E Every day

7.How often do you drink alcohol?
A Never B ≤1 time per month C 2-4 times per month D 2-3 times per week E 4 or more times per week

8.How often do you consume smoked food products (such as smoked bacon, sausages, etc.) in your daily life (excluding the Lunar New Year)?
A Never B Occasionally C Sometimes D Frequently E Every day

9.How often do you consume pickled food products (pickled vegetables, salted vegetables, pickled meat, etc.)?
A Never B Occasionally C Sometimes D Frequently E Every day

10.How often do you have hot pot in a month?
A ≤1 time B 2-3 times C 4-5 times D 6-7 times E ≥8 times

11.Do you consciously reduce the intake of cooking oil in your daily diet (e.g., using a limited amount of oil, consuming less fried food, etc.)?
A Never B Occasionally C Sometimes D Frequently E Every day

12.Do you consciously reduce the intake of salt in your daily diet (e.g., using a salt spoon, consuming a light diet, consuming fewer snacks,

etc.)?

A Never B Occasionally C Sometimes D Frequently E Every day

13.During the wild mushroom picking season, have you paid attention to news and knowledge related to their safety issues?

A Never B Occasionally C Sometimes D Frequently E Always

This questionnaire ends here. Thank you!
